# Aldehyde-Assisted Alkoxysilane Condensation to Form Siloxane Bond: A New Process for Curing Alkoxy-Functional Silicone Resins

**DOI:** 10.3390/molecules30030714

**Published:** 2025-02-05

**Authors:** Sławomir Rubinsztajn, Urszula Mizerska, Jan Kurjata, Małgorzata Kwiatkowska, Marek Cypryk

**Affiliations:** Centre of Molecular and Macromolecular Studies of Polish Academy of Sciences, 90-363 Lodz, Poland; urszula.mizerska@cbmm.lodz.pl (U.M.); jan.kurjata@cbmm.lodz.pl (J.K.); malgorzata.kwiatkowska@cbmm.lodz.pl (M.K.); marek.cypryk@cbmm.lodz.pl (M.C.)

**Keywords:** alkoxysilane, condensation, aldehyde, silicone resins, cationic Ge(II) compounds, siloxane bond

## Abstract

The formation of the siloxane bond is one of the most important reactions used in silicone chemistry and technology. In this paper, a new process for the condensation of alkoxy-functional silanes to form a siloxane bond is presented. The new reaction is catalyzed by a Ge(II)^+^ complex stabilized by pentamethylcyclopentadiene with a weakly coordinated anion, tetrakis(pentafluorophenyl)borane. A mechanistic study of this new condensation process using model alkoxy-functional silanes and propionaldehyde is completed. It is established that the quantitative conversion of alkoxysilanes to the siloxane bond requires stoichiometric amounts of aldehyde. It is also found that paraldehyde can serve as a convenient, higher boiling source of acetaldehyde for the condensation of alkoxysilanes. The results obtained, supported by DFT calculations, allow for us to formulate the mechanism of this reaction.

## 1. Introduction

Prof. Jutzi and his coworkers were the first to prepare divalent cationic Si(II) and Ge(II) compounds stabilized by the π-complex with pentamethylcyclopentadiene (Cp*) more than 20 years ago [1,2,3,4]. Initially considered eccentric, these compounds are now attracting increasing interest due to their potential catalytic properties in various important reactions [5,6]. Recently, the Wacker Chemie AG team led by Fritz-Langhals prepared several novel Cp*-stabilized Si(II)^+^ and Ge(II)^+^ complexes with weakly coordinated anions (WCA) and studied their catalytic properties in various reactions important for silicone technology [7,8,9]. They discovered that Cp*Si(II)^+^ B(C_6_F_5_)_4_^−^ and Cp*Ge(II)^+^ B(C_6_F_5_)_4_^−^ can be successfully used as catalysts in hydrosilylation of double and triple carbon–carbon bonds using a very low catalyst concentration, below 0.01 mol% [7,8,10]. This makes their catalytic activity comparable to the industrially used platinum compounds [11,12]. Moreover, these compounds are highly effective in promoting the coupling reaction between hydrosilanes and alkoxysilanes, known as the Piers–Rubinsztajn reaction [7,9]. They also catalyze the oxidative coupling of hydrosilanes in the presence of stoichiometric amounts of aldehydes or ketones, leading to the formation of a siloxane bond and dialkyl ether as a by-product [8,9]. The proposed mechanism of this process involves the coordination of two alkoxysilane molecules, which is formed in the first step of this process, to a cationic Si(II)^+^ or Ge(II)^+^ center. The formation of the siloxane bond and the ether is facilitated in such a complex [8]. This new coupling process was successfully applied to the synthesis of phenyl siloxane copolymers [8,9]. If such a complex between Cp*Mt(II)^+^ and two alkoxysilane molecules, where Mt = Si or Ge, is indeed formed, direct condensation of alkoxysilanes to a disiloxane and a dialkyl ether should be possible. Cp*Ge(II)^+^ WCA^−^ catalysts exhibit much better stability towards oxygen and moisture than their Si(II) analogs, which allows for their use under typical laboratory conditions [8]. Cp*Ge(II)^+^ B(C_6_F_5_)_4_^−^ proved to be particularly attractive for commercial applications due to its relatively simple synthesis and the commercial availability of the starting materials. Recent patent applications by the WACKER’s team claim that Cp*Ge(II)^+^ B(C_6_F_5_)_4_^−^ catalyzes the crosslinking of the alkoxy-functional silicone resins [13,14]. Interestingly, the presence of a carbonyl compound is required for this process to occur. The mechanism of this new condensation reaction is unknown. In this article, we present a detailed study of this reaction, which led us to formulate the mechanism of this new condensation process.

## 2. Results and Discussion

### 2.1. Study of the Curing Process of the Alkoxy-Functional Siloxane Resins

Recently filed patent applications by Wacker Chemie AG claim that Cp*Ge(II)^+^ B(C_6_F_5_)_4_^−^ is an effective catalyst for the crosslinking of alkoxy-functional siloxane resins in the absence of moisture. It was also claimed that the addition of about 10 to 20 mol%, based on the alkoxy content, of carbonyl compounds, such as aldehydes or ketones, facilitates the new condensation process [13,14]. The mechanism of this new and commercially attractive curing process is unknown. Detailed mechanistic studies were initiated to gain knowledge about this new condensation process.

Screening experiments of the curing process of methoxy-functional silicone resin (Resin 1) were completed. Resin 1 is a liquid silicone resin produced by hydrolytic condensation of the MeSi(OMe)_3_. It contains approximately 31 wt.% of the methoxy groups [13,14]. The curing process of Resin 1 was examined under various conditions as its 50 wt.% solution in dry dichloromethane in the presence of 0.09 wt.% of Cp*Ge(II)^+^ B(C_6_F_5_)_4_^−^. The results of the study are summarized in Table 1.

The catalyzed mixture of Resin 1 is stable for several days when stored in an atmosphere of dry nitrogen or dry oxygen. However, exposure of the catalyzed Resin 1 mixture to humid air (RH = 74%) or humid nitrogen (RH = 90%) causes it to gel (Table 1, Exp. 2 and 3). Similar results were obtained in analogous experiments with the ethoxy-functional silicone resin (Resin 2), which is formed by the hydrolytic condensation of MeSi(OEt)_3_ [13,14] (Table S1, Exp. 2 and 3). The ethoxy group content in Resin 2 is approximately 41 wt.%. These observations indicate that the presence of moisture is necessary for curing the alkoxy-functional silicone resins. Obviously, due to the presence of the Si-OMe group, Resin 1 gels faster than Resin 2, which has ethoxy groups attached to the polymer chain. Thus, the cationic complex of Germanium (II), Cp*Ge(II)^+^ B(C_6_F_5_)_4_^−^ appears to be an active catalyst for the widely used silicone technology process of crosslinking alkoxy-functional silicone polymers in the presence of moisture [11,15].

As mentioned earlier, recent patent applications from WACKER claim that the addition of approximately 10% by weight acetaldehyde is necessary for the rapid cure of Resin 1 in the presence of Cp*Ge(II)^+^ B(C_6_F_5_)_4_^−^. The formation of a singlet at σ = 3.2 ppm, which was assigned to dimethyl ether, was observed in a separate ^1^H NMR experiment under these conditions (see Example 10 in both patent applications) [13,14]. The generation of dimethyl ether may suggest direct coupling of the SiOMe groups, leading to the formation of a disiloxane bond, which is catalyzed by the acetaldehyde—Cp*Ge(II)^+^ B(C_6_F_5_)_4_^−^ complex. However, it should be noted that the examples presented in these patent applications do not explicitly mention that the curing process was carried out in a moisture-free atmosphere. Thus, a crosslinking process involving hydrolysis and condensation of Si-OR groups cannot be excluded.

**Figure 1:**
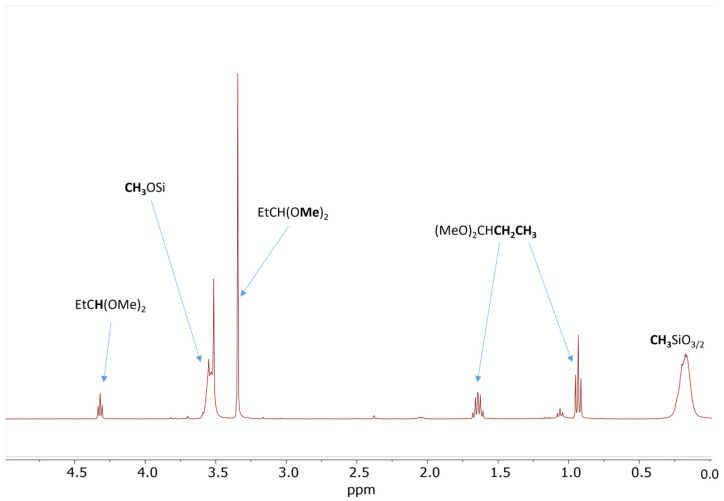
^1^H NMR spectrum of the 50 wt% solution of Resin 1 in DCM catalyzed by 0.09 wt.% of Cp*Ge(II)^+^ B(C_6_F_5_)_4_^−^ after addition of 18 mol% of propionaldehyde. The spectrum was recorded after 5 min of reaction.

The results of the screening experiments with the addition of propionaldehyde confirmed that, indeed, about 14 mol% propionaldehyde relative to SiOMe groups is necessary for the gelation of Resin 1 under anhydrous conditions (Table 1, Experiments 5–8). The ^1^H NMR spectrum recorded after 5 min of reaction with 18 mol% of propionaldehyde showed about 37% conversion of SiOMe groups and formation of a sharp singlet at σ = 3.34 ppm (Figure 1). There are three additional signals associated with this singlet: a triplet at σ = 0.93 ppm, a multiplet at σ = 1.64 ppm, and a triplet at σ = 4.32 ppm. The observed signals and their integrations clearly show that the major product of this reaction is propionaldehyde dimethyl acetal. Similar ^1^H NMR spectra were recorded after the addition of 5 mol%, 14 mol%, and 24 mol% propionaldehyde (Figures S1–S3). The formation of propionaldehyde dimethyl acetal was also independently confirmed by gas chromatography–mass spectroscopy (GC/MS) analysis (Figure S4).

A similar effect of propionaldehyde addition was observed in the case of Resin 2, but the time to gel formation was correspondingly longer (Table S1). The ^1^H NMR spectrum of the catalyzed Resin 2 recorded 30 min after the addition of 41 mol% propionaldehyde indicates the conversion of approximately 65% of the SiOEt groups and the formation of the corresponding propionaldehyde diethyl acetal (Figure S5). It is worth mentioning that the two sets of multiplets centered at 3.5 ppm and 3.65 ppm are assigned to the methylene protons of the -CH(OCH_2_CH_3_)_2_ group. The complex splitting pattern of the methylene protons is related to the diastereotopic nature of these protons in the formed diethyl acetal [16]. The presence of propionaldehyde diethyl acetal was also independently confirmed by gas chromatography–mass spectroscopy (GC/MS) analysis (Figure S6). The Resin 2 solution gelled after an additional 50 min (80 min total) of reaction at these conditions.

**Scheme 1:**
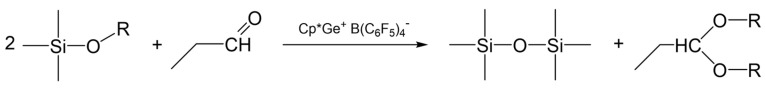
The proposed curing process of the alkoxy-functional siloxanes in the presence of propionaldehyde.

The results obtained from the screening experiments suggest that the curing process in the presence of propionaldehyde must involve de-alkoxylation reactions leading to the formation of a siloxane bond and propionaldehyde dialkyl acetal as a by-product (Scheme 1).

### 2.2. Model Study of the SiOR + SiOR Condensation Reaction Catalyzed by Cp*Ge^+^ B(C_6_F_5_)_4_^−^

It is important to gain basic knowledge about the above-presented new condensation reaction of siloxanes with alkoxy-functional groups catalyzed by Cp*Ge^+^B(C_6_F_5_)_4_^−^ in the presence of aldehyde, which leads to the formation of a new siloxane bond with the release of the corresponding dialkyl acetal as a by-product. For this reason, studies of this process were carried out using model alkoxysilanes, such as PhMe_2_SiOMe and PhMe_2_SiOEt, and the simplest alkoxysiloxane, 1,1,1,2,2-pentamethylmethoxydisiloxane (MM^OMe^). ^1^H NMR and ^29^Si NMR spectroscopy and GC/MS were used as the main analytical techniques to identify substrate conversion and product formation.

**Figure 2:**
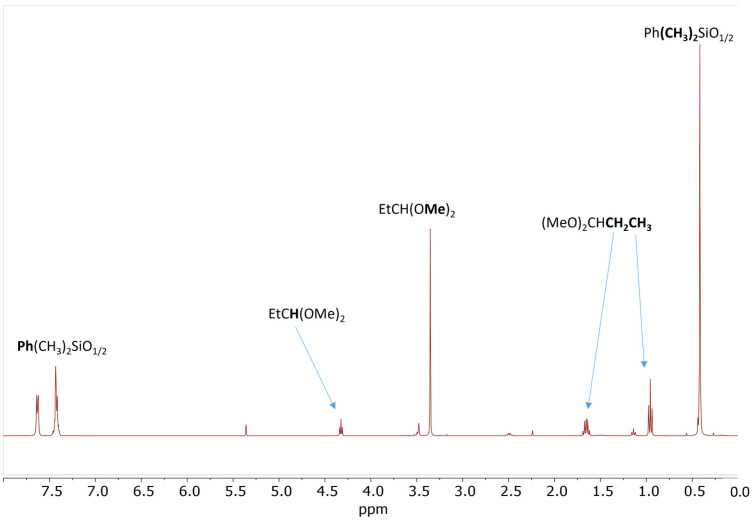
^1^H NMR spectrum of the reaction mixture consisting of 0.56 mol/L PhMe_2_SiOMe and 0.28 mol/L propionaldehyde in the presence of 0.5 mol% Cp*Ge^+^ B(C_6_F_5_)_4_^−^ recorded after 30 min of reaction.

**Figure 3:**
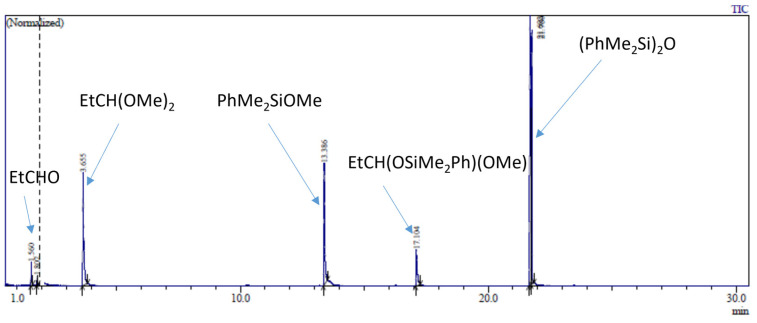
GC/MS analysis of the reaction mixture consisting of 0.56 mol/L PhMe_2_SiOMe, 0.28 mol/L propionaldehyde, and 0.5 mol% of Cp*Ge^+^ B(C_6_F_5_)_4_^−^ completed after 5 min of the reaction.

The reaction of 20 eq. of Me_2_PhSiOMe with 10 eq. of propionaldehyde in the presence of 0.5 mol% Cp*Ge^+^B(C_6_F_5_)_4_^−^ was studied in CD_2_Cl_2_ using ^1^H NMR spectroscopy. The rapid conversion of alkoxysilane to diphenyltetramethyldisiloxane after 30 min of reaction was observed at these conditions. The ^1^H NMR spectrum clearly showed that the formation of disiloxane is accompanied by the quantitative formation of propionaldehyde dimethyl acetal (Figure 2). The dimethyl ether signal at σ = 3.2 ppm was not present in the recorded spectrum. The presence of dimethyl acetal was also confirmed by GC/MS. The GC/MS analysis performed approximately 5 min after mixing of all reagents with the catalyst showed the presence of the starting alkoxysilane and aldehyde as well as the disiloxane product and propionaldehyde dimethyl acetal (Figure 3 and Figure S7). An additional GC signal was detected at RT = 17.1 min. Its MS fragmentation pattern consists of a mixed acetal, EtCH(OMe)(OSiPhMe_2_). This silylated acetal is most likely the product of the first step of the de-alkoxylation condensation process involving the reaction of an alkoxysilane with propionaldehyde. The mixed acetal formed is likely to be highly reactive and will quickly react with a second alkoxysilane molecule in a subsequent step, resulting in the formation of the final disiloxane and dimethyl acetal. The quantitative formation of diphenyltetramethyldisiloxane was independently confirmed by ^29^Si NMR spectroscopy (Figure S8).

**Figure 4:**
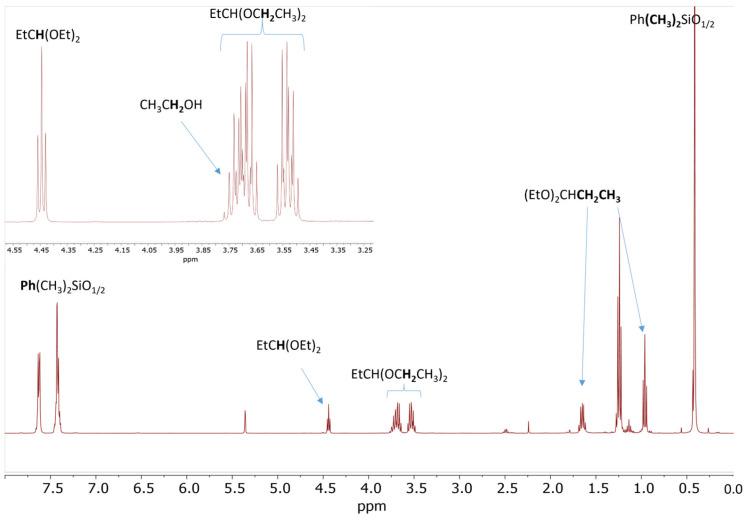
^1^H NMR spectrum of the reaction mixture consisting of 0.56 mol/L PhMe_2_SiOEt and 0.28 mol/L propionaldehyde in the presence of 0.5 mol% Cp*Ge^+^ B(C_6_F_5_)_4_^−^ recorded after 60 min of reaction.

A similar process was observed for the reaction of 20 eq. PhMe_2_SiOEt and 10 eq. propionaldehyde in the presence of 0.5 mol% Cp*Ge^+^ B(C_6_F_5_)_4_^−^. The ^1^H NMR spectrum recorded after 60 min of reaction indicates approximately 90% conversion of PhMe_2_OEt to the corresponding disiloxane and the quantitative formation of propionaldehyde diethyl acetal as a by-product (Figure 4).

**Scheme 2:**
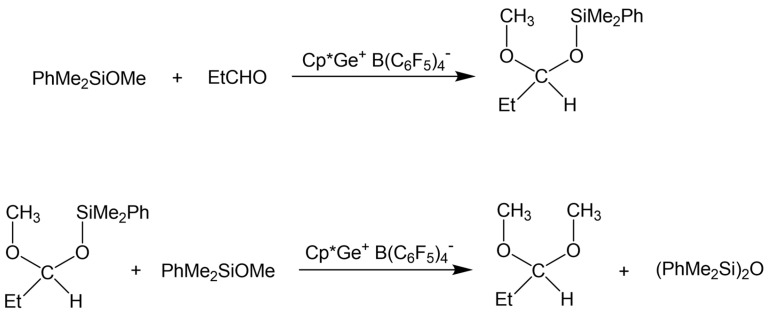
Proposed two-step de-alkoxylation condensation of alkoxysilanes in the presence of a stoichiometric amount of aldehyde.

The above results revealed that the de-alkoxylation condensation of alkoxysilanes in the presence of stoichiometric amounts of aldehyde catalyzed by Cp*Ge^+^ B(C_6_F_5_)_4_^−^ is a two-step process (Scheme 2), where the first step involves the addition of alkoxysilane to the aldehyde, resulting in the formation of a mixed silylated acetal. The next step must be a reaction between this intermediate and a second alkoxysilane molecule, producing a disiloxane and a symmetrical dialkyl acetal.

The analogous reaction of trimethylmethoxysilane with various carbonyl compounds catalyzed by 1 mol% trimethylsilyl trifluoromethanesulfonate was already reported by Noyori in 1980 as a convenient method for acetal synthesis [17]. Subsequently, Sakurai described the efficient acetalization of carbonyl compounds with tetramethoxysilane catalyzed by 5 mol% of iodotrimethylsilane [18]. However, the mechanism of these reactions has not been studied in detail. Interestingly, there are no research studies reported in the available literature describing the application of this type of reaction to the synthesis of siloxane materials.

**Figure 5:**
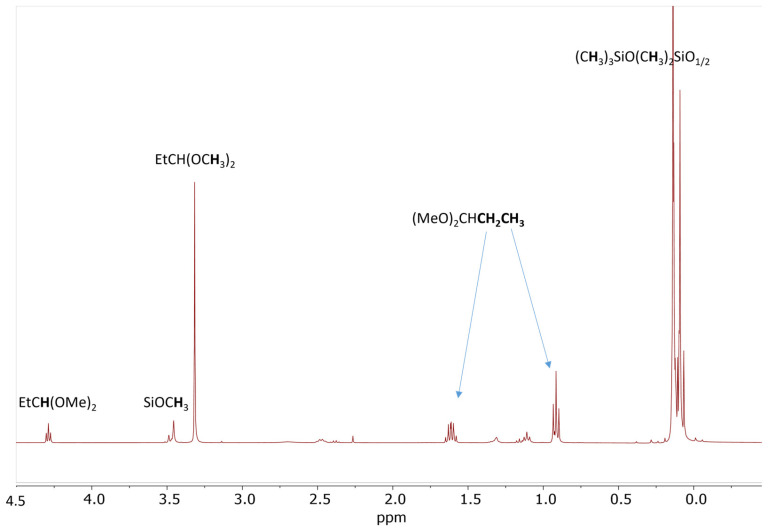
^1^H NMR spectrum of the reaction mixture consisting of 0.56 mol/L MM^OMe^, 0.28 mol/L propionaldehyde, and 0.5 mol% Cp*Ge^+^ B(C_6_F_5_)_4_^−^ recorded after 5 min of reaction.

Model studies of de-alkoxylation condensation in the presence of aldehyde were extended to pentamethylmethoxydisiloxane, MM^OMe^, which is the simplest model of a siloxane with an alkoxy-functional group. A reaction mixture of 20 eq. MM^OMe^ with 10 eq. propionaldehyde reacts quickly after introducing 0.5 mol% Cp*Ge^+^ B(C_6_F_5_)_4_^−^ in anhydrous DCM. The ^1^H NMR spectrum recorded 5 min after the addition of the catalyst shows over 80% conversion of the SiOMe groups and the formation of an appropriate amount of propionaldehyde dimethyl acetal as a by-product (Figure 5).

**Figure 6:**
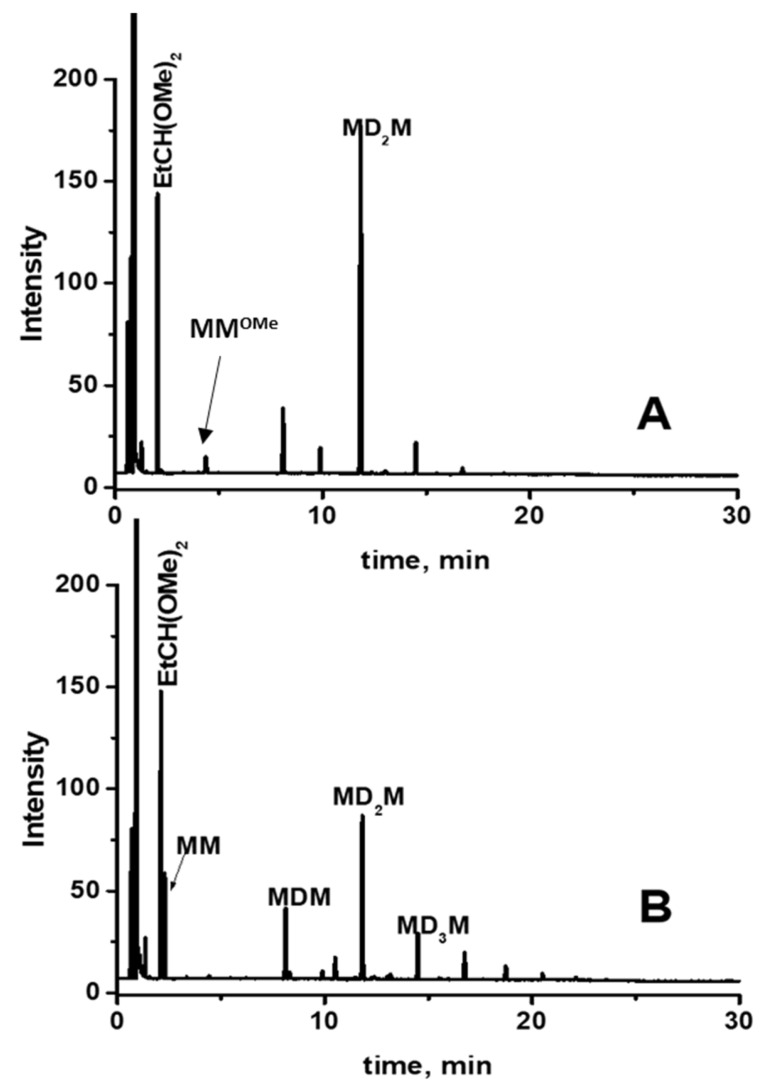
GC analysis of the reaction mixture consisting of 0.56 mol/L MM^OMe^, 0.28 mol/L propionaldehyde, and 0.5 mol% Cp*Ge^+^ B(C_6_F_5_)_4_^−^ recorded (**A**) after 5 min and (**B**) after 60 min of reaction.

**Figure 7:**
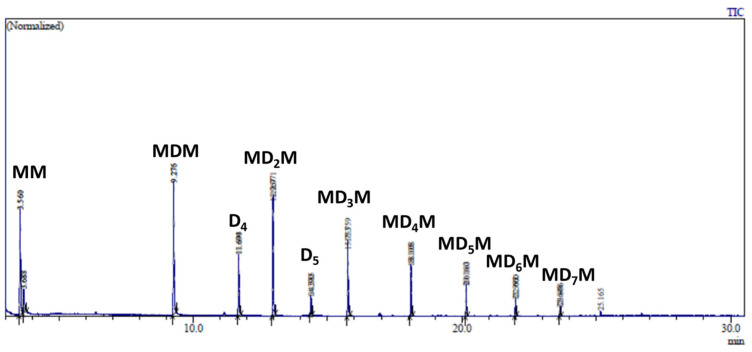
GC/MS analysis of the reaction mixture consisting of 0.56 mol/L MM^OMe^, 0.28 mol/L propionaldehyde, and 0.5 mol% Cp*Ge^+^ B(C_6_F_5_)_4_^−^ recorded after 24 h of reaction.

GC analysis of this reaction mixture after 5 min of reaction indicates the formation of decamethyltetrasiloxane (MD_2_M) as the major product (Figure 6A). The GC signal of propionaldehyde dimethyl acetal was also observed. However, subsequent analysis after 60 min of reaction shows the formation of several trimethylsilyl-terminated oligosiloxanes (MD_n_M, where n = 0, 1, 2…) (Figure 6B). The formation of trimethylsilyl-terminated oligosiloxanes was confirmed by GC/MS analysis of the reaction mixture performed after 24 h, which showed the presence of MD_n_M linear oligomers and cyclic oligomers D_4_ and D_5_ (Figure 7 and Figure S9).

**Scheme 3:**
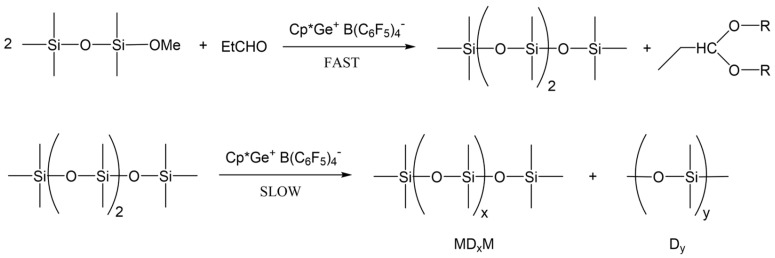
De-alkoxylation reaction of MM^OMe^ and a subsequent redistribution process of MD_2_M.

These results reveal that the de-alkoxylation condensation of MM^OMe^ in the presence of a stoichiometric amount of propionaldehyde leading to linear decamethyltetrasiloxane (MD_2_M) is the fastest reaction. However, the resulting MD_2_M product undergoes a subsequent siloxane redistribution reaction to the various cyclic (D_y_) and linear oligosiloxanes (MD_x_M). This siloxane redistribution reaction is also catalyzed by Cp*Ge^+^ B(C_6_F_5_)_4_^−^. Cp*Ge^+^ B(C_6_F_5_)_4_^−^ must act as a strong Lewis acid capable of activating the siloxane bond to participate in the redistribution reaction of siloxane units (Scheme 3) [19,20].

### 2.3. Study of the SiOR + SiOR Condensation Reaction in the Presence of Paraldehyde Catalyzed by Cp*Ge BArF_4_

**Scheme 4:**
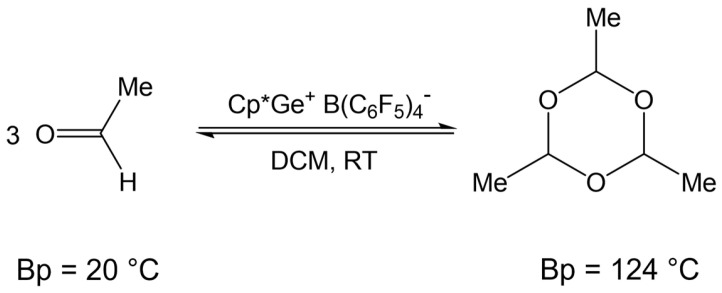
Reversible cyclotrimerization of acetaldehyde catalyzed by Cp*Mt^+^ B(C_6_F_5_)_4_^−^, where Mt = Si(II) or Ge(II).

Fritz-Langhals recently reported that Cp*Si^+^ B(C_6_F_5_)_4_^−^ and Cp*Ge^+^ B(C_6_F_5_)_4_^−^ are effective catalysts for the reversible cyclotrimerization of aldehydes such as acetaldehyde (Scheme 4) [8]. She demonstrated that paraldehyde, having a boiling point of 124 °C, can serve as a convenient source of volatile acetaldehyde in the oxidative coupling of hydrosilanes catalyzed by Cp*Ge^+^ B(C_6_F_5_)_4_^−^.

**Figure 8:**
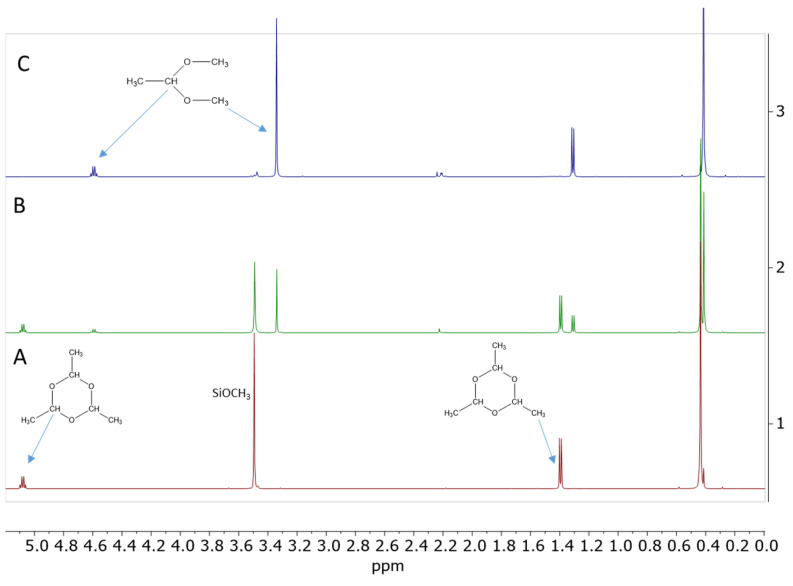
^1^H NMR spectra of the reaction mixture consisting of 0.56 mol/L PhMe_2_SiOMe with 0.09 mol/L paraldehyde in the presence of 0.0028 mol/L Cp*Ge^+^ B(C_6_F_5_)_4_^−^ recorded (**A**) before the addition of the catalyst, (**B**) t = 2 min, and (**C**) t = 60 min after the addition of the catalyst.

**Figure 9:**
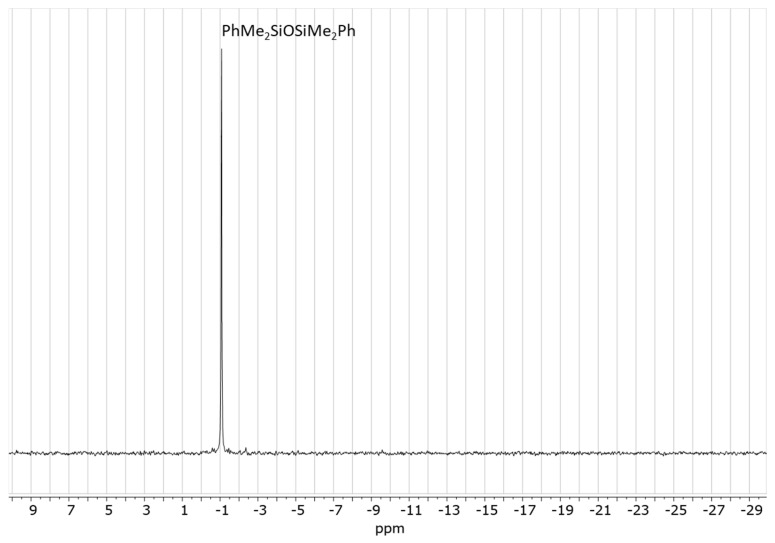
^29^Si NMR spectrum of the reaction mixture consisting of 0.56 mol/L PhMe_2_SiOMe with 0.09 mol/L paraldehyde in the presence of 0.0028 mol/L Cp*Ge^+^ B(C_6_F_5_)_4_^−^ recorded after 24 h.

It was tested whether paraldehyde could play the same role in the de-alkoxylation condensation process. A reaction mixture consisting of 20 eq. (0.56 mol/L) PhMe_2_SiOMe and 3.3 eq. (0.09 mol/L) paraldehyde in DCM was prepared. ^1^H NMR spectrum showed that the alkoxysilane did not react with paraldehyde under these conditions. However, upon the addition of 0.1 eq. (0.0028 mol/L) Cp*Ge^+^ B(C_6_F_5_)_4_^−^, a very rapid de-alkoxylation reaction occurred, which led to the complete conversion of PhMe_2_SiOMe to the corresponding disiloxane and the formation of acetaldehyde dimethyl acetal as a by-product in less than 1 h (Figure 8). The formation of the disiloxane was confirmed by ^29^Si NMR (Figure 9).

The conversion of PhMe_2_SiOEt in the presence of paraldehyde was also tested under the same conditions. As expected, the de-alkoxylation reaction of phenyl(dimethyl)ethoxysilane is much slower than that of phenyl(dimethyl)methoxysilane. It takes about 6 h to achieve 100% conversion of ethoxysilane to the corresponding disiloxane and acetaldehyde diethyl acetal at these conditions (Figure S10).

The above results confirmed that Cp*Ge^+^ B(C_6_F_5_)_4_^−^ is an effective catalyst for the reversible decomposition of paraldehyde to acetaldehyde and that the formed acetaldehyde can participate in the de-alkoxylation reaction, which is promoted by the same catalyst. Thus, paraldehyde, having a relatively high boiling point, can serve as a convenient source of acetaldehyde for the de-alkoxylation condensation of alkoxysilanes catalyzed by Cp*Ge^+^ B(C_6_F_5_)_4_^−^.

### 2.4. Testing Decomposition of Dialkyl Acetal in the Presence of Cp*Ge^+^ B(C_6_F_5_)_4_^−^

**Scheme 5:**
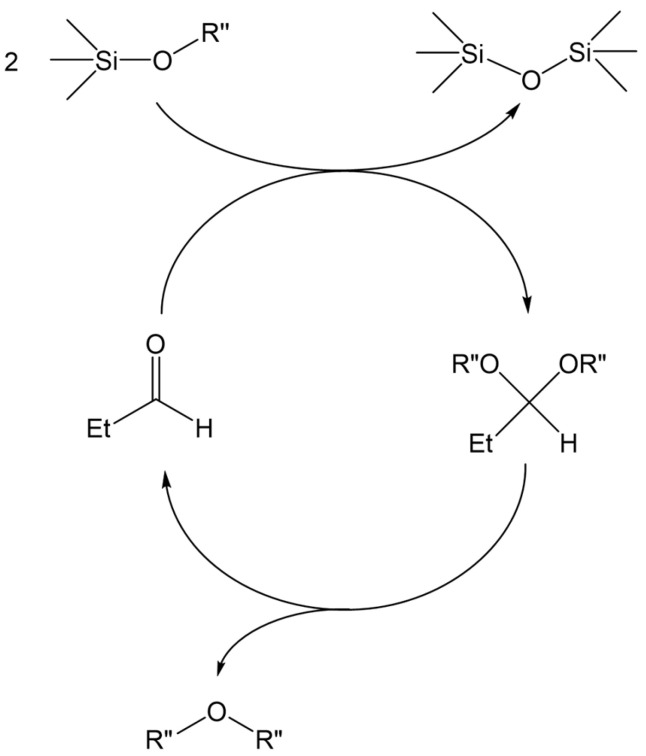
The hypothetical mechanism of the coupling of SiOR groups to SiOSi in the presence of sub-stoichiometric amounts of aldehyde.

It is possible that the de-alkoxylation reaction of alkoxy-functional silanes can lead to the quantitative formation of disiloxane in a catalytic cycle involving sub-stoichiometric amounts of aldehyde (Scheme 5). Such a hypothetical mechanism must involve the decomposition of the formed dialkyl acetal to its corresponding aldehyde and the release of the dialkyl ether as a by-product.

**Scheme 6:**
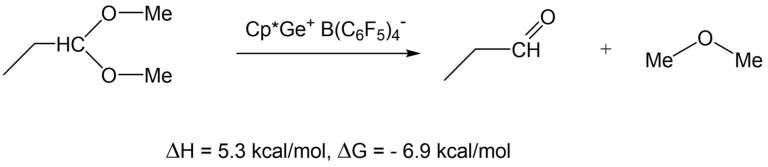
DFT calculation of the hypothetical decomposition of dimethyl acetal of propionaldehyde.

DFT calculations have shown that such a reaction is thermodynamically favorable (ΔG = −6.9 kcal/mol) (Scheme 6). However, to the best of our knowledge, there is no evidence in the literature for such a decomposition reaction to occur.

**Figure 10:**
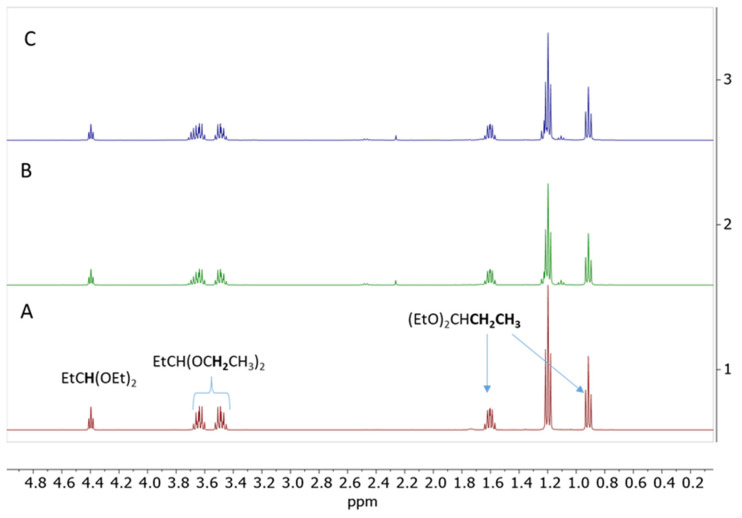
^1^H NMR spectrum of propionaldehyde diethyl acetal in the presence of 1 mol% of Cp*Ge^+^ B(C_6_F_5_)_4_^−^ recorded at (**A**)—t = 0 min, (**B**)—t = 5 min, and (**C**)—t = 24 h of reaction at RT.

The potential decomposition of propionaldehyde diethyl acetal was evaluated at RT in the presence of 1 mol% of Cp*Ge^+^ B(C_6_F_5_)_4_^−^. The reaction was monitored by ^1^H NMR. This simple experiment provided no evidence of the decomposition of propionaldehyde diethyl acetal to diethyl ether and propionaldehyde, even after 24 h at room temperature (Figure 10).

Based on the above results, the de-alkoxylation condensation of alkoxysilanes to siloxane in the presence of a sub-stoichiometric amount of aldehyde, accompanied by the formation of dialkyl ether and recovery of aldehyde, can be excluded.

### 2.5. Mechanism of the De-Alkoxylation Reaction in the Presence of Aldehyde—DFT Calculations

The experimental results obtained support the tentative mechanism of the de-alkoxylation reaction in the presence of aldehyde (Scheme 2). In order to gain a deeper understanding of this process, DFT calculations were conducted.

To save computational time, the calculations were performed using the simpler CpGe^+^ model catalyst, where Cp is cyclopentadiene, instead of Cp*Ge^+^. It is reasonable to assume that the mechanism of catalysis by both Germanium species is very similar, as suggested by some preliminary calculations. It was also assumed that the influence of the low-nucleophilic B(C_6_F_5_)_4_^−^ counterion, which interacts only weakly with electrophiles, on the course of the reaction is not significant and may be neglected. Furthermore, the calculations were performed for gas phase conditions due to the significant errors that implicit solvation models can introduce in estimating the free energies of stationary points. Of course, these simplifications limit the accuracy of the calculations. For example, one may expect that interaction with the counterion might slightly lower the free energy barriers of the reaction. On the other hand, solvation by a polar solvent may somewhat favor the increased dissociation of intermediate species.

The enthalpy and free energy of the interaction of CpGe^+^ and Cp*Ge^+^ with selected nucleophiles, which are reactants of interest, are presented below (Table 2).

A comparison of the nucleophilicity (ΔG of complex formation) of the given nucleophiles toward Ge^+^(II) Lewis acids in the gas phase reveals that methoxysilane and propionaldehyde have comparable nucleophilicity, and the weakest nucleophile is disiloxane. The calculated ΔG values for Cp*Ge^+^ are more positive than for the simple CpGe^+^, which suggests lower acidity of the Cp*Ge^+^ Lewis acid. This may be due to the inductive electronic effect of Me groups resulting in a reduction in the positive charge on the Ge center.

**Scheme 7:**
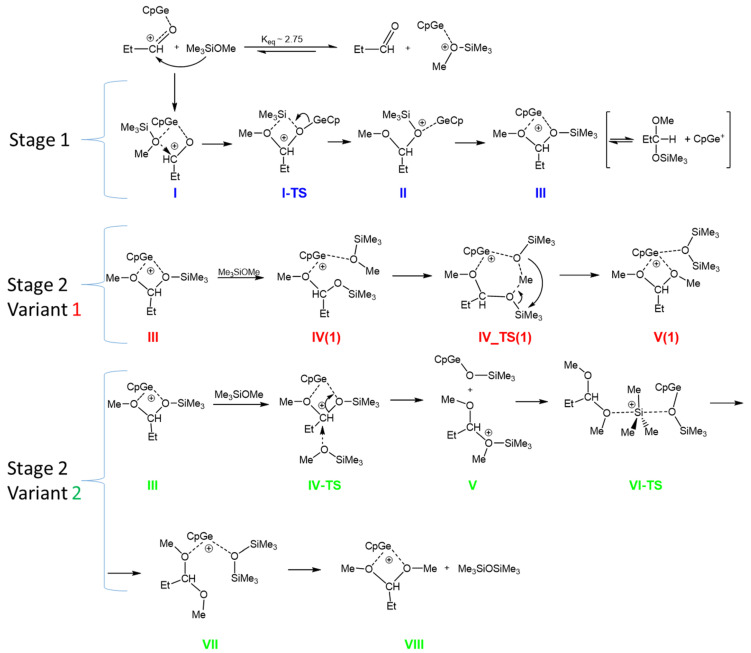
The hypothetical mechanism of the de-alkoxylation reaction in the gas phase. Blue—1st stage, Red—variant 1 of the 2nd stage, Green—variant 2 of the 2nd stage.

Based on these results, the equilibrium constant for the formation of the complexes between CpGe^+^ with EtCHO and MeOSiMe_3_ in the gas phase was estimated to be K_eq_ = 2.75, as shown in Scheme 7. The above calculations show that CpGe^+^ interacts with Me_3_SiOMe slightly stronger than with propionaldehyde. Thus, the concentration of the CpGe^+^--MeOSiMe_3_ complex is about three times higher than the CpGe^+^ complex with aldehyde. However, in the case of Cp*Ge^+^, the complex with propionaldehyde dominates at approximately a 5/1 ratio. This implies that, statistically, both reaction pathways are possible: one involving the nucleophilic attack of the carbonyl group on the Si center of the alkoxysilane, which is complexed with a cationic Ge(II) compound, and the other involving the nucleophilic attack of the alkoxysilane on the carbonyl carbon, which is complexed with the same Ge(II)^+^ compound. However, we must remember that the reaction mechanism, including the direct nucleophilic attack of a carbonyl oxygen on an electrophilic center (other than a proton), is very rare in organic chemistry [20]. Therefore, it was decided to investigate the mechanism of the de-alkoxylation process, which involves the generally accepted in the literature attack of a nucleophile, in this case, an alkoxysilane, on the carbon center of the carbonyl group [21] activated by the cationic Ge(II) compound (Scheme 7, Stage 1).

Stage 1 involves formation of the complex in which CpGe^+^ is coordinated to both EtCHO and MeOSiMe_3_ (**I**). This complex facilitates the attack of methoxysilane on carbonyl carbon in aldehyde with the transfer of the trimethylsilyl group to the aldehyde oxygen (**I-TS**). This produces mixed methylsilyl acetal complexed with CpGe^+^ (**II**), which isomerizes to the more stable form (**III**). Mixed methoxysiloxy acetal is apparently higher in energy than the substrates propionaldehyde + methoxysilane (Table 3). However, this step (reversible dissociation of the complex **III** to mixed acetal and CpGe^+^) may be neglected if the subsequent attack of the second molecule of MeOSiMe_3_ proceeds directly on **III**.

In the mixed acetal complex **III**, there are two electrophilic centers: a silicon center and a carbonyl carbon. Both possible pathways of the second stage were investigated by DFT calculations: the nucleophilic attack of the alkoxysilane on the silicon center (Variant 1) and the nucleophilic attack of the alkoxysilane on the carbonyl carbon (Variant 2) (Scheme 7).

The calculated stationary points found for the 2nd stage (Variant 1) of this process, involving the nucleophilic attack of MeOSiMe_3_ oxygen on silicon in a mixed acetal, are shown in Scheme 7. However, the calculated energy barriers are very high (ΔG^‡^ of ca. 50 kcal/mol), which makes this reaction path unlikely. For this reason, the mechanism involving the 1st variant of the 2nd stage of the de-alkoxylation process was excluded. The more detailed data concerning this pathway, the structures, energies, and the free energy profile for this variant are presented in Supplementary Information (Table S2, Figures S11 and S12).

Therefore, we focused on Variant 2 of Stage 2 in which the attack of methoxysilane oxygen proceeds on the carbonyl carbon in the acetal molecule complexed with CpGe cation (**III**). This attack results in dissociation of the complex (structure **V**). Subsequent reorientation of the silyloxonium fragment makes possible the attack of the silyloxy-germanium fragment on silicon in the silyloxonium cation, which consequently leads to hexamethyldisiloxane and dimethyl acetal complexed with CpGe^+^, as shown in structure **VII**. Eventually, complex **VII** can split to release disiloxane (**VIII**).

**Figure 11:**
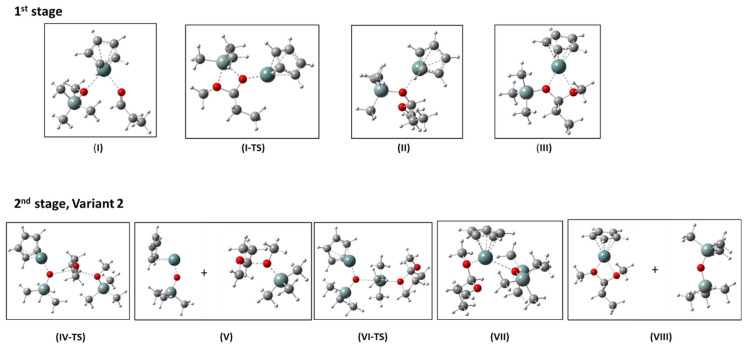
Calculated structures of the stationary points **I**, **II**, and **III** and the transition state, **I-TS**, (1st stage), and **IV-TS**, **V**, **VI-TS**, **VII**, **VIII** (2nd stage, Variant 2) of the de-alkoxylation reaction catalyzed by CpGe^+^. Colors of elements: white—H, grey—C, steel-grey—Si, darker steel-grey—Ge, red—O.

**Figure 12:**
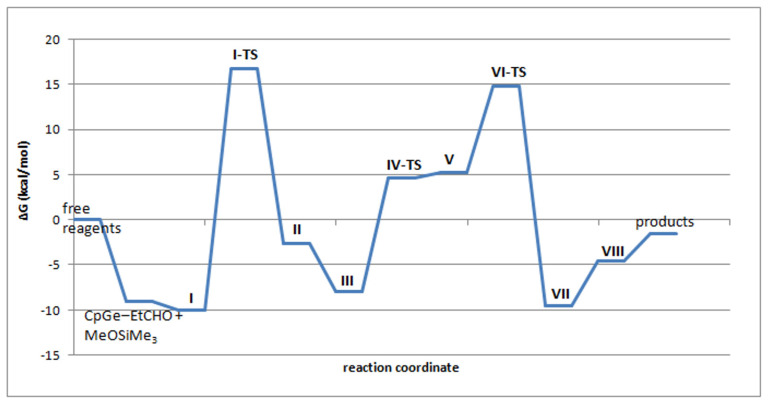
Calculated free energy profile of the reaction of Me_3_SiOMe with EtCHO catalyzed by CpGe^+^ in the gas phase.

The structures as well as relative enthalpies and free energies of the stationary points (**I-VIII**) along the reaction path of the proposed mechanism for the de-alkoxylation reaction involving Stage 2 Variant 2 are presented in Figure 11 and Table 3. Figure 12 presents the free energy profile of the reaction according to the discussed mechanism.

## 3. Materials and Methods

### 3.1. Materials

Phenyldimethylsilane (Fluorochem, Hadfield, UK, 98%), diphenylsilane (ABCR, Karlsruhe, Germany, 97%), 1,1,1, 3, 3-pentamethyldisiloxane (ABCR, Karlsruhe, Germany, 97%), 1,1-diethoxypropane (Aldrich, Darmstadt, Germany, 97%), and paraldehyde (Aldrich, Darmstadt, Germany, 97%) were used without further purification. Propionaldehyde (Aldrich, Darmstadt, Germany, 97%) was dried and purified by distillation. The 5% Pd/C was purchased from Aldrich (Darmstadt, Germany). The Cp*Ge^+^ B(C_6_F_5_)_4_^−^ catalyst and alkoxy-functional silicone resins (Resin 1 and Resin 2) were obtained from WACKER (Munich, Germany) and were used without further purification. The solvents DCM, Et_2_O, hexane, toluene, and THF were dried using standard procedures.

### 3.2. Synthesis of Alkoxysilanes and Siloxanes

Model alkoxy-functional silanes and siloxanes were prepared using a similar procedure presented below for dimethylphenylethoxysilane (PhMe_2_SiOEt). A total of 8.89 g (0.064 mol) of PhMe_2_SiH, 8 mL of hexane, and 0.131 g of 5% Pd/C were introduced into a round-bottomed flask equipped with a reflux condenser and addition funnel. The reaction was carried out in an atmosphere of iert gas (argon). Then, 7.24 g (0.156 mol) of EtOH in 7 mL of hexane was added dropwise to the reaction mixture. After 15 min, GC analysis was performed, which showed complete conversion of the silane. The mixture was then filtered through celite to remove the catalyst. Hexane was evaporated using a water aspirator, leaving behind 8.28 g (74.02% yield). GC analysis showed the formation of PhMe_2_SiOEt and complete conversion of the substrate. The crude reaction product was distilled using a small Vigreux distillation column, yielding 7.95 g of approximately 95% pure by GC of PhMe_2_SiOEt, boiling point at 180 °C/15 mmHg.

### 3.3. NMR Spectroscopy

^1^H, ^13^C, and ^29^Si NMR spectra in CDCl_3_ and CD_2_Cl_2_ were obtained with a Bruker 400 MHz spectrometer operated at 100.58 and 79.46 MHz, respectively. ^29^Si NMR spectra were recorded with broadband proton decoupling. A heteronuclear gated decoupling with 20 s delay technique was used to acquire ^29^Si NMR spectra.

### 3.4. Gas Chromatography–Mass Spectrometry (GC/MS)

GC/MS analysis was performed using a Shimadzu QP2010 ultra-apparatus equipped with a Zebron ZB-5MSi Capillary GC Column (30 m × 0.25 mm × 0.25 μm). The carrier gas was helium. The following temperature program was used: hold at 50 °C for 3 min, heat to 250 °C at a rate of 10 °C/min, hold at 250 °C for 20 min, heat to 280 °C at a rate 20 °C/min. Quadrupole mass spectrometer, Shimadzu QP2010 Ultra, with electron ionization was connected to a GC system.

### 3.5. Gas Chromatography

Gas chromatography analyses were performed using an Agilent 8860 GC System (Agilent Technologies, Inc., Santa Clara, CA, USA) equipped with a thermal conductivity detector and an HP-1 capillary column (Agilent Technologies, Inc., Santa Clara, CA, USA) with a diameter of 0.53 mm and a length of 30 m. The carrier gas was helium, flow rate was 5 mL/min, detector temperature was 250 °C, injector temperature was 250 °C, and column temperature program was as follows: hold for 5 min at 40 °C isotherm, heat to 240 °C at a rate of 10 °C/min.

### 3.6. Study of the Condensation Cure of the Alkoxy-Functional Silicone Resins

A solution of 50% by weight alkoxy-functional silicone resin in dry DCM was placed in a small vial equipped with a magnetic stirrer. The vial was placed inside a small, controlled atmosphere reactor. After establishing the equilibrium state, the desired amount of catalyst solution was introduced using a hypodermic syringe. The behavior of the resin solution was observed visually. The gelation time was determined when the rotation of the magnetic stirrer stopped. Small samples of approximately 10 µL of the resin solution were taken at various time intervals, introduced into 500 µL of deuterated DCM, and analyzed by ^1^H NMR to assess conversion of the alkoxy groups.

### 3.7. Study of the Condensation of Model Alkoxysilanes in the Presence of Propionaldehyde

A total of 58 mg (20 eq.) of PhMe_2_SiOMe and 10.1 mg (10 eq.) of propionaldehyde were placed into an NMR tube with 500 µL of deuterated DCM. The 1st NMR spectrum was recorded. The desired amount (1 or 0.1 eq.) of Cp*Ge^+^B(C_6_F_5_)_4_^−^ solution in DCM was added, and next, the ^1^H NMR spectrum was collected (time—approximately 5 min). The additional ^1^H NMR spectra were collected after 30 min and 1, 3, 6, and 24 h. In some experiments, the additional samples were taken and analyzed by GCMS.

### 3.8. Theoretical Methods

All quantum mechanical calculations were performed using the Gaussian 16 suite of programs [22]. Geometries of the bases and base pair model systems were optimized using the hybrid B3LYP density functional [23] corrected for dispersion interactions using Grimme D3 empirical term with Becke-Johnson damping [24,25], with Def2TZVP basis set in the gas phase. This level of theory is denoted as B3LYP-D3BJ/Def2TZVP. The Def2xxVP family of basis sets was chosen because it covers a wide range of elements up to Rn and was proven to give consistent and reliable results [26]. All stationary points were identified as stable minima or transition states by frequency calculations. The vibrational analysis provided thermal enthalpy and entropy corrections at 298 K within the rigid rotor/harmonic oscillator/ideal gas approximation [22]. Thermochemical corrections were scaled by a factor of 0.985 (https://comp.chem.umn.edu/freqscale/version3b2.htm (accessed on 25 January 2025)). The integration grid was set to ultrafine. Solvation effect was estimated using the implicit solvation model (Conductor-like Polarizable Continuum Model, CPCM) [27]. The basis set superimposition error (BSSE) was neglected since the method of its estimation [28] is not accurate enough to precisely calculate weak interactions.

## 4. Conclusions

The curing process of alkoxy-functional silicone resins in the presence of catalytic amounts of Cp*Ge^+^ B(C_6_F_5_)_4_^−^ was studied. It was found that the catalyzed mixtures of these resins are stable in an atmosphere of dry nitrogen and dry oxygen for several days. However, exposure of the catalyzed mixtures to moisture causes their gelation in less than 24 h. Thus, the curing process of alkoxy-functional silicone resins in the presence of Cp*Ge^+^ B(C_6_F_5_)_4_^−^ involves hydrolysis and condensation of alkoxy groups under these conditions. The addition of propionaldehyde to the catalyzed mixture of alkoxy-functional silicone resins causes their gelation even under anhydrous conditions. The formation of dialkyl acetal as a by-product was confirmed by ^1^H NMR and GCMS. The study of the new condensation reaction, which we call de-alkoxylation condensation, was extended to model mono alkoxy-functional silanes. It was found that mono alkoxy-functional silanes are rapidly and quantitatively converted to the corresponding disiloxane and dialkyl acetal as a by-product in the presence of stoichiometric amounts of aldehyde via a two-step reaction involving the formation of a mixed acetal as an intermediate. Based on the results obtained and DFT calculations, a mechanism for the de-alkoxylation condensation was proposed involving a nucleophilic attack of the alkoxysilane on the carbonyl carbon of the aldehyde–Cp*Ge^+^ B(C_6_F_5_)_4_^−^ complex in the first step. This mixed acetal complex with Cp*Ge^+^ B(C_6_F_5_)_4_^−^ undergoes a rapid nucleophilic substitution reaction on the acetal carbon in the second step, leading to the formation of a dialkyl acetal and disiloxane. Furthermore, it was found that paraldehyde can serve as a convenient aldehyde source for the de-alkoxylation reaction of alkoxysilanes in the presence of Cp*Ge^+^ B(C_6_F_5_)_4_^−^.

## Figures and Tables

**Table 1:** Results of screening tests of the curing process of Resin 1 at 25 °C in the presence of 0.09 wt% Cp*Ge(II)^+^ B(C_6_F_5_)_4_^−^.

Experiment #	Atmosphere	Propionaldehyde mol% vs. SiOMe	Time to Gel
1	Dry nitrogen	none	Liquid after 1 week
2	Air, RH = 74%	none	8 h
3	Nitrogen, RH = 90%	none	4 h
4	Dry oxygen	none	Liquid after 1 week
5	Dry nitrogen	5	Liquid after 1 week
6	Dry nitrogen	14	1.5 h
7	Dry nitrogen	18	20 min
8	Dry nitrogen	24	10 min

**Table 2:** Enthalpy and free energy (Kcal/mol) of the interaction of CpGe^+^ and Cp*Ge^+^ with selected nucleophiles in the gas phase.

	ΔH	ΔG
**CpGe^+^---Nu**		
EtCHO	−16.6	−9.1
MeOSiMe_3_	−18.9	−9.7
Me_3_SiOSiMe_3_	−17.5	−5.3
Cp*Ge^+^---Nu		
EtCHO	−12.7	−4.9
MeOSiMe_3_	−14.0	−4.0
Me_3_SiOSiMe_3_	−12.6	−0.7

**Table 3:** Relative enthalpies and free energies (Kcal/mol) of stationary points along the reaction path of the proposed mechanism (The sum of the energies of free reagents was arbitrarily set to zero).

	ΔH_rel_	ΔG_rel_
CpGe^+^ + EtCHO + MeOSiMe_3_	0	0
CpGe^+^–EtCHO + MeOSiMe_3_	−16.6	−9.1
CpGe^+^–MeOSiMe_3_ + EtCHO	−18.9	−9.7
**I**	−30.7	−10.0
**I-TS**	−8.5	16.7
**II**	−26.1	−2.6
**III**	−31.6	−8.0
CpGe^+^ + EtCH(OMe)(OSiMe_3_)	−8.3	5.3
**IV-TS**	−30.9	4.6
**V**	−29.8	5.9
**VI**	−19.7	5.2
**VI-TS**	−24.6	14.8
**VII**	−46.5	−9.5
**VIII,** CpGe^+^–EtCHO + Me_3_SiOSiMe_3_	−19.4	−4.6
CpGe^+^ + EtCH(OMe)_2_ + Me_3_SiOSiMe_3_	−14.9	−1.6

## Data Availability

The data presented in this study are available on request from the corresponding author.

## Supplementary Materials

The following supporting information can be downloaded at: https://www.mdpi.com/article/10.3390/molecules30030714/s1. **Table S1**. Results of screening tests of the curing process of Resin 2 at 25 °C in the presence of 0.09 wt% Cp*Ge(II)^+^ B(C_6_F_5_)_4_^−^. **Table S2.** DFT calculated relative enthalpies and free energies (Kcal/mol) of stationary points along the reaction path of the mechanism (2nd Stage, Variant 1) involving an attack on the Si center. **Figure S1**. ^1^H NMR spectrum of the 50 wt% solution of Resin 1 in DCM catalyzed by 0.09 wt% of Cp*Ge(II)^+^ B(C_6_F_5_)_4_^−^ after addition of 5 mol% of propionaldehyde. **Figure S2**. ^1^H NMR spectrum of the 50 wt% solution of Resin 1 in DCM catalyzed by 0.09 wt% of Cp*Ge(II)^+^ B(C_6_F_5_)_4_^−^ after addition of 14 mol% of propionaldehyde. **Figure S3**. ^1^H NMR spectrum of the 50 wt% solution of Resin 1 in DCM catalyzed by 0.09 wt% of Cp*Ge(II)^+^ B(C_6_F_5_)_4_^−^ after addition of 24 mol% of propionaldehyde. **Figure S4.** GCMS analysis of 50wt % solution of Resin 1 in DCM catalyzed by 0.09 wt% of Cp*Ge(II)^+^ B(C_6_F_5_)_4_^−^ after addition of 40 mol% of propionaldehyde. **Figure S5.** ^1^H NMR spectrum of the 50wt % solution of Resin 2 in DCM catalyzed by 0.09 wt% of Cp*Ge(II)^+^ B(C_6_F_5_)_4_^−^ after addition of 40 mol% of propionaldehyde. **Figure S6**. GCMS analysis of 50 wt % solution of Resin 2 in DCM catalyzed by 0.09 wt% of Cp*Ge(II)^+^ B(C_6_F_5_)_4_^−^ after addition of 40 mol% of propionaldehyde. **Figure S7**. MS fragmentation of the GC/MS signals of the reaction mixture consisting of 0.56 mol/L PhMe_2_SiOMe, 0.28 mol/L propionaldehyde and 0.5 mol% of Cp*Ge^+^ B(C_6_F_5_)_4_^−^ completed after 5 min of the reaction. **Figure S8.** ^29^Si NMR spectrum of the reaction mixture consisting of 0.56 mol/L PhMe_2_SiOMe, 0.28 mol/L propionaldehyde in the presence of 0.5 mol% Cp*Ge^+^ B(C_6_F_5_)_4_^−^ recorded after 24 h of reaction. **Figure S9**. MS fragmentation of the GC/MS signals of the reaction mixture consisting of 0.56 mol/L MM^OMe^, 0.28 mol/L propionaldehyde, and 0.5 mol% Cp*Ge^+^ B(C_6_F_5_)_4_^−^ recorded after 24 h of reaction. **Figure S10.** ^1^H NMR spectra of the reaction mixture consisting of 0.56 mol/L of PhMe_2_SiOEt with 0.09 mol/L paraldehyde in the presence of 0.0028 mol/L Cp*Ge^+^ B(C_6_F_5_)_4_^−^ recorded before the addition of the catalyst, 2 min, 60 min, and 360 min after the addition of the catalyst. **Figure S11.** Calculated structures of the stationary points IV(1) and V(1) and the transition state, IV-TS(1), (2nd stage, Variant 1) of the de-alkoxylation reaction catalyzed by CpGe^+^. Colors of elements: white—H, grey—C, steel-grey—Si, darker steel-grey—Ge, red—O. **Figure S12.** Calculated relative enthalpies and free energies (Kcal/mol) of stationary points along the reaction path of the mechanism involving the attack on Si center (2nd Stage, Variant 1).
