# Supplementary material for: Aldehyde-Assisted Alkoxysilane Condensation to Form Siloxane Bond: A New Process for Curing Alkoxy-Functional Silicone Resins
Source: Molecules. 2025 Feb 5;30(3):714. doi: 10.3390/molecules30030714 (PMC11820733; doi:10.3390/molecules30030714)
Supplement: Supplementary file 1 [file molecules-30-00714-s001.zip › molecules-3441436-supplementary.pdf]

Supplemental Information

Aldehyde-Assisted Siloxane Bond Formation via Coupling of Alkoxysilanes. A New Process for Curing Alkoxy-functional Silicone Resins.

Sławomir Rubinsztajn, Urszula Mizerska, Jan Kurjata, Małgorzata Kwiatkowska and Marek Cypryk

Centre of Molecular and Macromolecular Studies of Polish Academy of Sciences, Lodz, Poland

Table S1. Results of screening tests of the curing process of Resin 2 at 25 °C in the presence of 0.09 wt% $Cp^{*}Ge(II)^{+}B(C_6F_5)_4^{-}$.

Experiment #	Atmosphere	Propionaldehyde mol% vs. SiOEt	Time to gel
1	Dry nitrogen	none	Liquid after 1 week
2	Air, RH = 74%	none	13 h
3	Nitrogen, RH = 90%	none	8 h
4	Dry oxygen	none	Liquid after 1 week
5	Dry nitrogen	12	Liquid after 1 week
6	Dry nitrogen	20	Liquid after 1 week
7	Dry nitrogen	31	2 h 44 min
8	Dry nitrogen	41	1 h 20 min

Table S2. DFT calculated relative enthalpies and free energies (Kcal/mol) of stationary points along the reaction path of the mechanism (2nd Stage, Variant 1) involving an attack on the Si center. (Sum of the energies of free reagents, $CpGe^{+}$, EtCHO, 2 $MeOSiMe_3$, was arbitrarily set to zero)

	ΔH_{rel}	ΔG_{rel}
**III** + $MeOSiMe_3$	-45.6	-8.0
**IV(1)**	-70.6	-38.6
**IV-TS(1)**	20.3	58.4
**V(1)**	-30.3	-5.5
$CpGe^{+}$ + $EtCH(OMe)_2$ + $Me_3SiOSiMe_3$	-14.9	-1.6

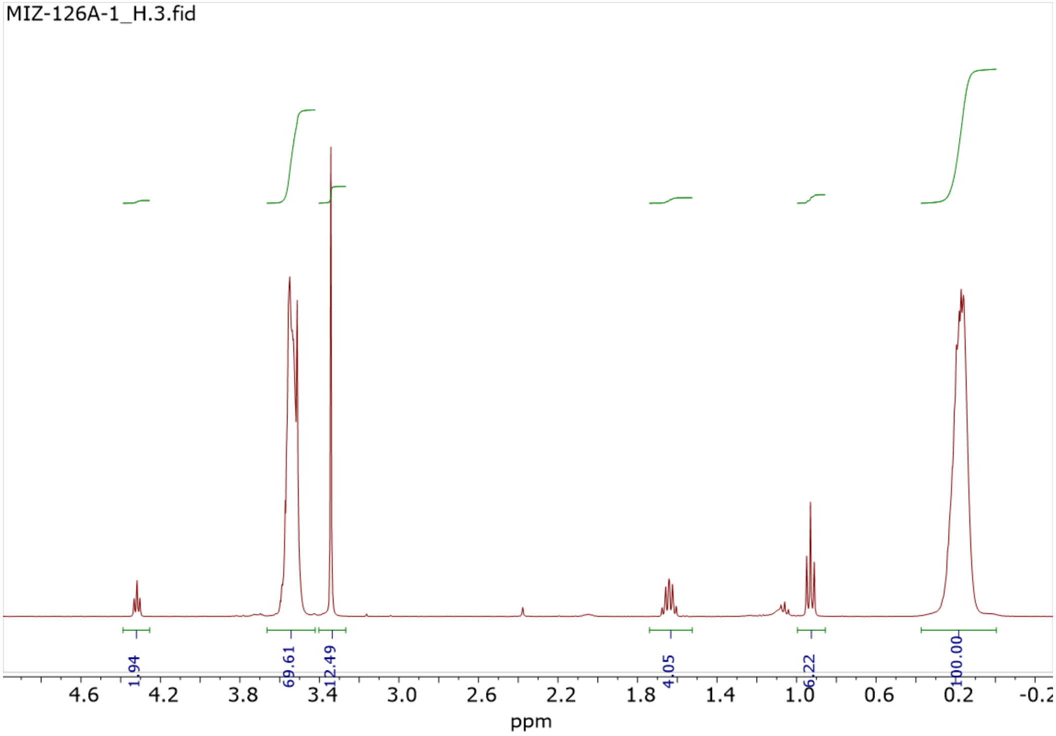

Figure S1. ^{1}H NMR spectrum of the 50 wt% solution of Resin 1 in DCM catalyzed by 0.09 wt% of $Cp^{*}Ge(II)^{+}$ $B(C_6F_5)_4^{-}$ after addition of 5 mol% of propionaldehyde. The spectrum was recorded after 60 min of reaction.

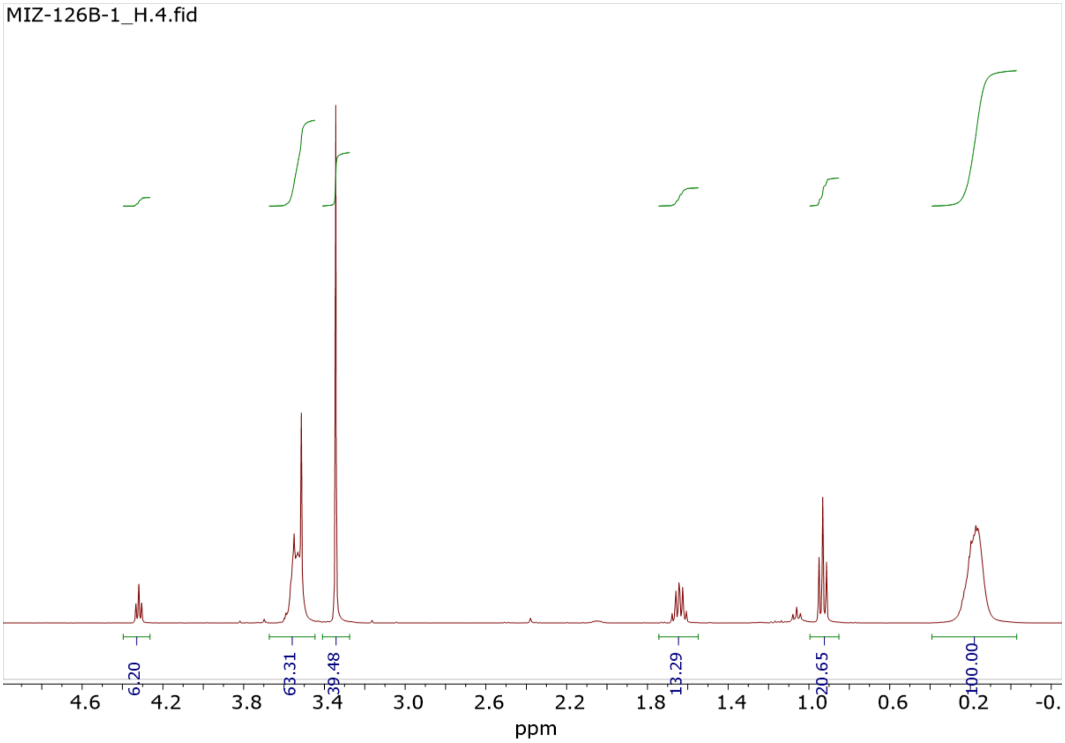

Figure S2. ^{1}H NMR spectrum of the 50 wt% solution of Resin 1 in DCM catalyzed by 0.09 wt% of $Cp^{*}Ge(II)^{+}$ $B(C_6F_5)_4^{-}$ after addition of 14 mol% of propionaldehyde. The spectrum was recorded after 5 min of reaction.

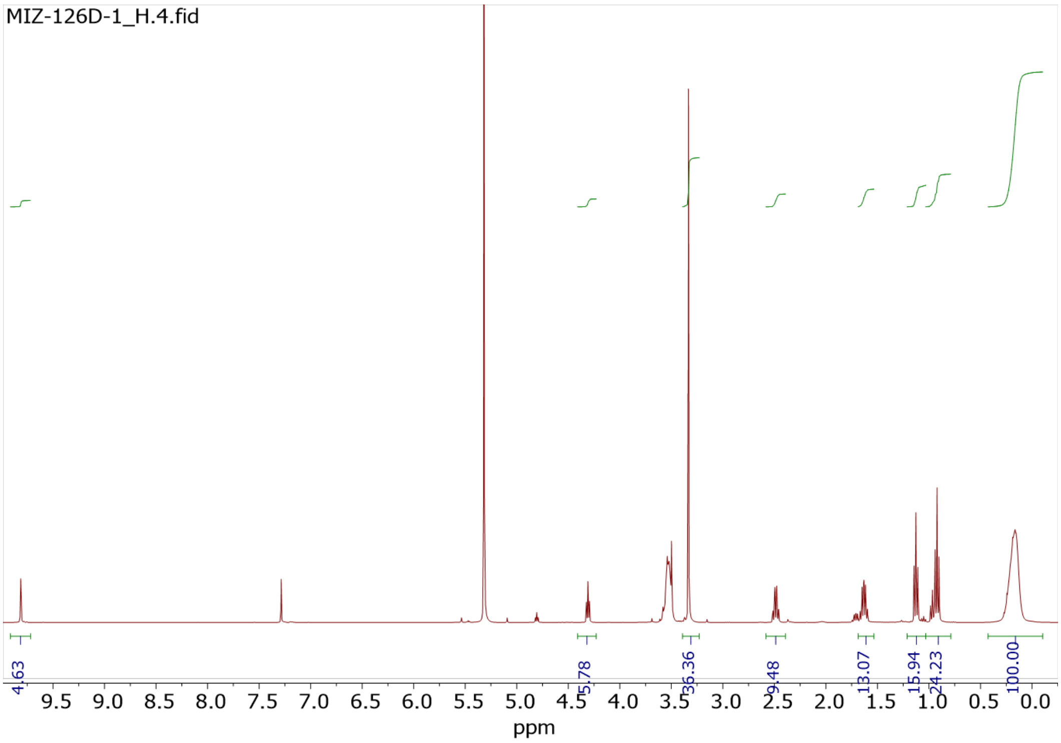

Figure S3. ^{1}H NMR spectrum of the 50 wt% solution of Resin 1 in DCM catalyzed by 0.09 wt% of Cp*Ge(II)$^+$ B(C_6F_5)$_4^-$ after addition of 24 mol% of propionaldehyde. The spectrum was recorded after 5 min of reaction.

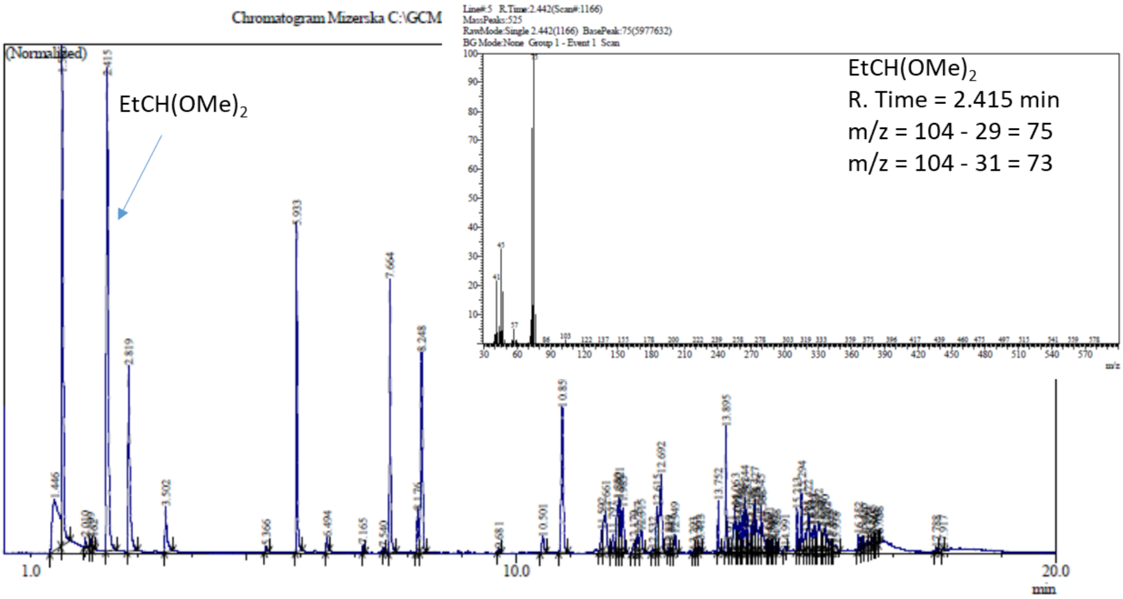

Figure S4. GCMS analysis of 50wt % solution of Resin 1 in DCM catalyzed by 0.09 wt% of Cp*Ge(II)$^+$ B(C_6F_5)$_4^-$ after addition of 40 mol% of propionaldehyde. GCMS analysis was completed after 30 min of reaction.

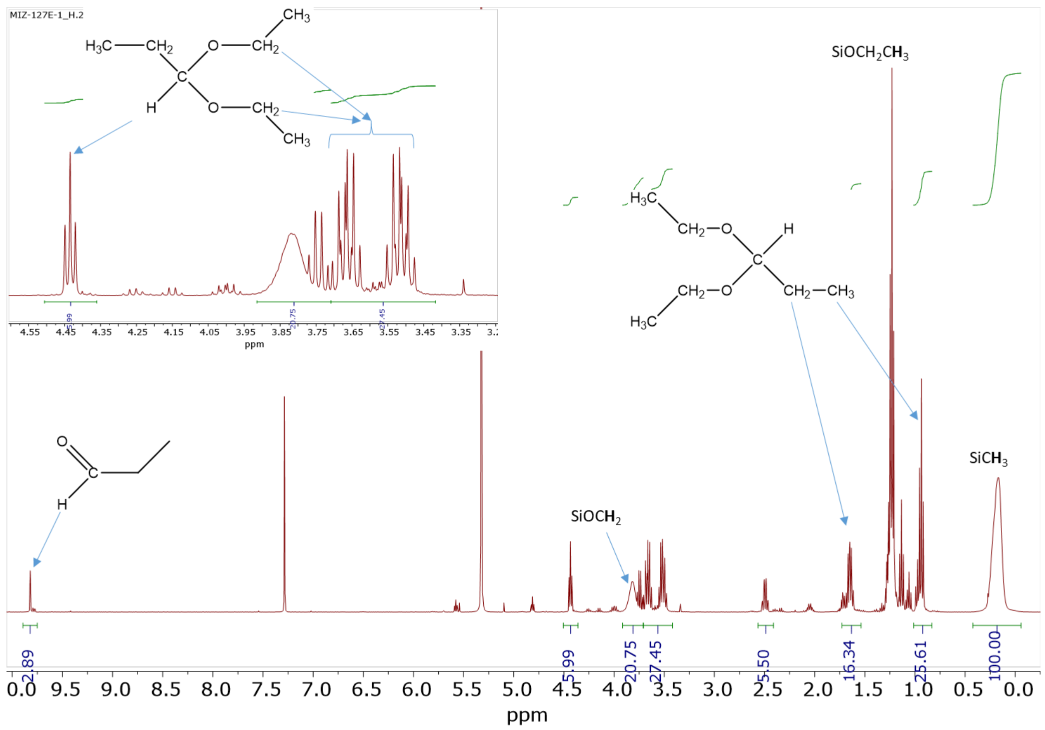

Figure S5. ^{1}H NMR spectrum of the 50wt % solution of Resin 2 in DCM catalyzed by 0.09 wt% of Cp*Ge(II)$^{+}$ $B(C_6F_5)_4^-$ after addition of 40 mol% of propionaldehyde. The spectrum was recorded after 30 min of reaction.

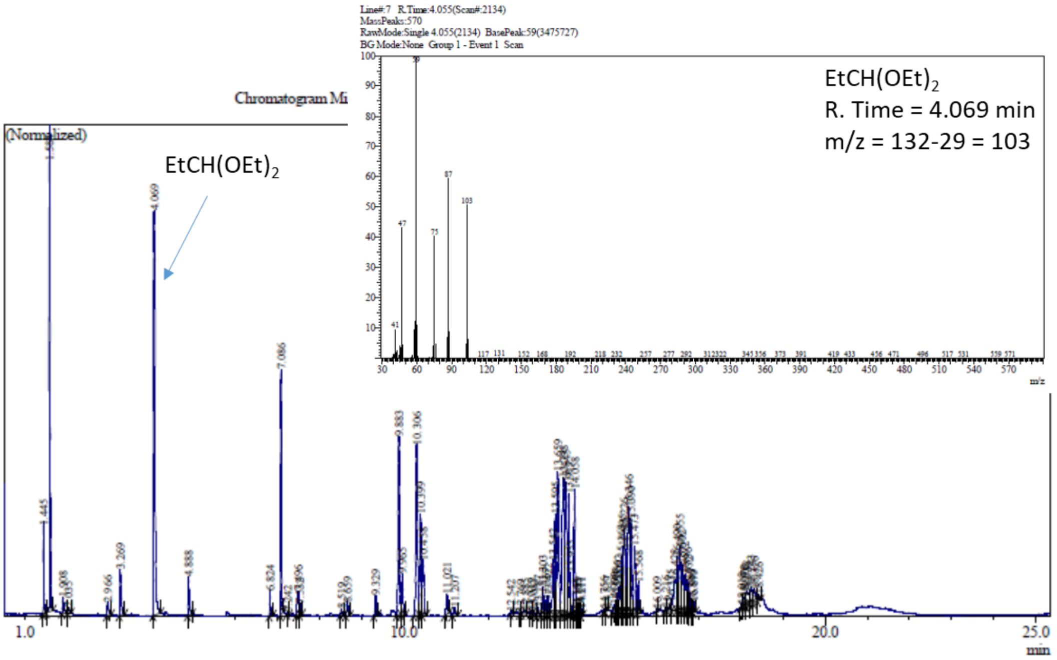

Figure S6. GCMS analysis of 50 wt % solution of Resin 2 in DCM catalyzed by 0.09 wt% of Cp*Ge(II)$^{+}$ $B(C_6F_5)_4^-$ after addition of 40 mol% of propionaldehyde.

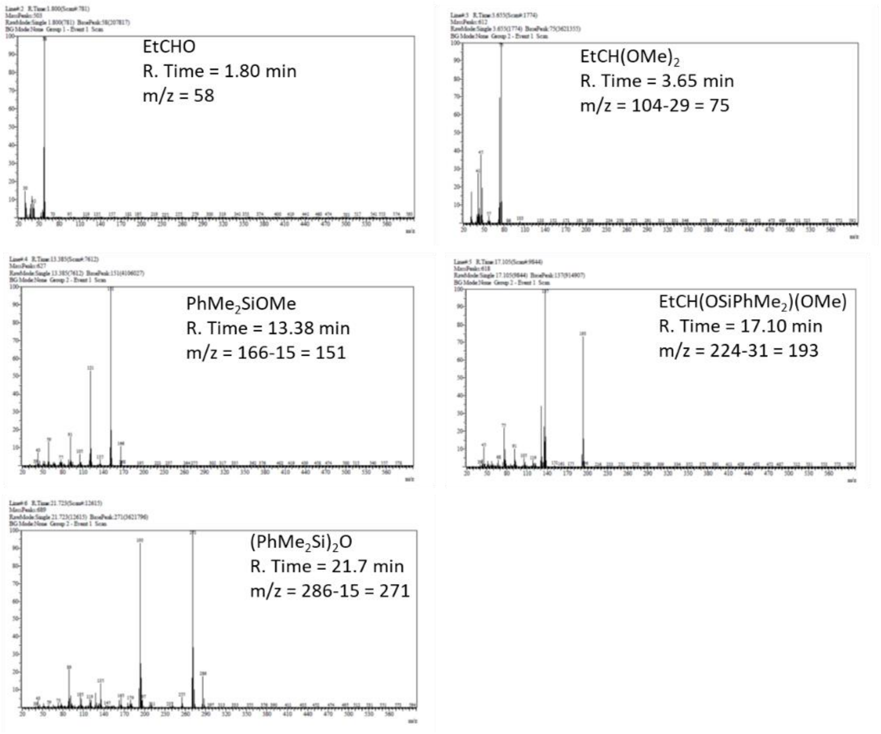

Figure S7. MS fragmentation of the GC/MS signals of the reaction mixture consisting of 0.56 mol/L $PhMe_2SiOMe$, 0.28 mol/L propionaldehyde and 0.5 mol% of $Cp^{*}Ge^{+}$ $B(C_6F_5)_4^{-}$ completed after 5 min of the reaction.

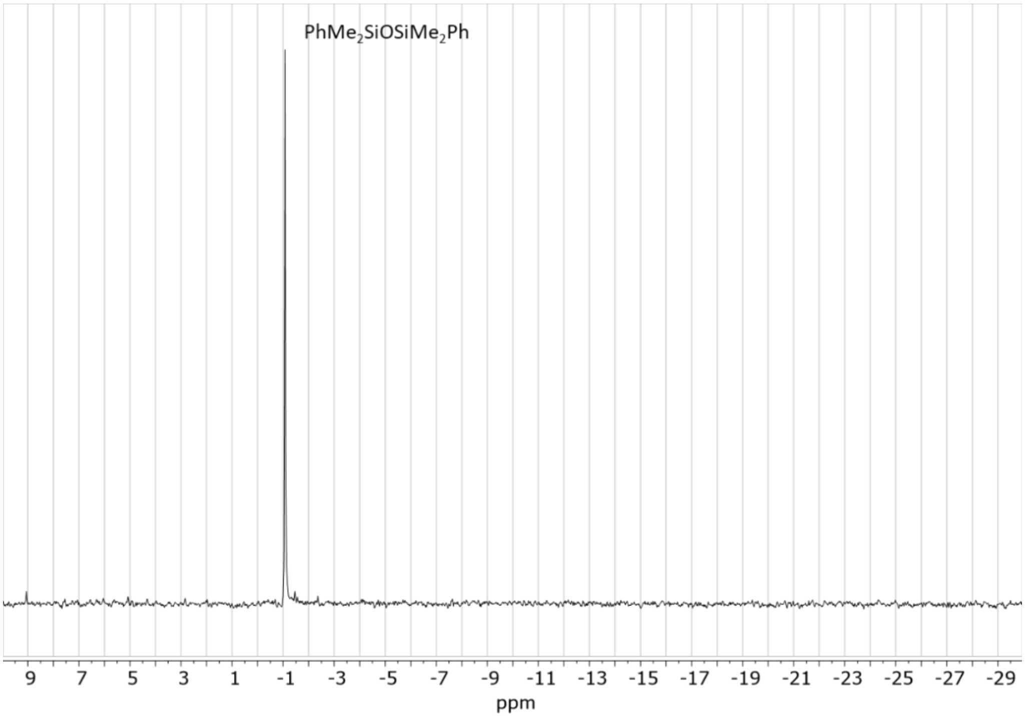

Figure S8. ^{29}Si NMR spectrum of the reaction mixture consisting of 0.56 mol/L $PhMe_2SiOMe$, 0.28 mol/L propionaldehyde in the presence of 0.5 mol% $Cp^{*}Ge^{+}$ $B(C_6F_5)_4^{-}$ recorded after 24 h of reaction.

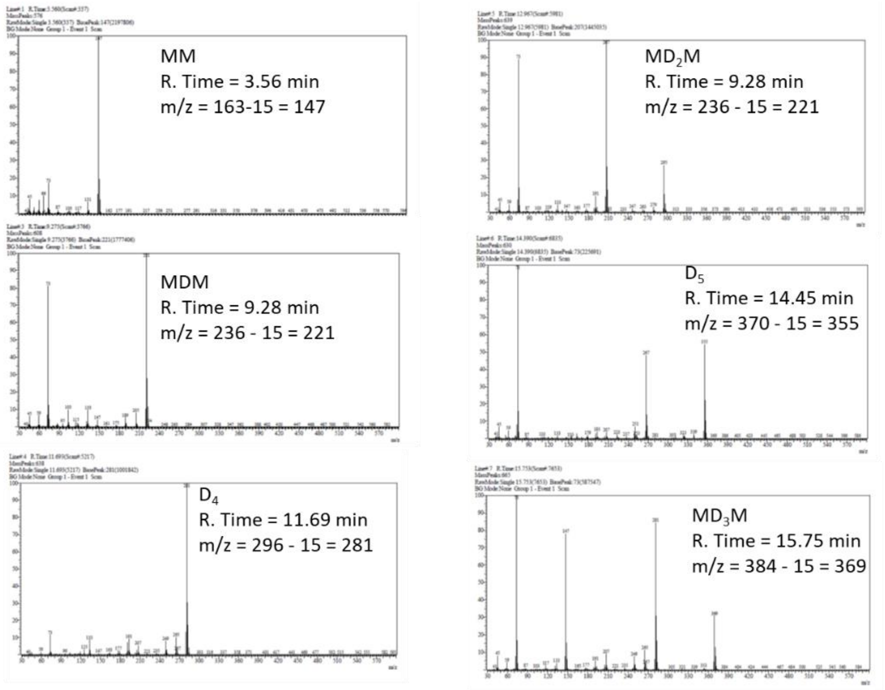

Figure S9. MS fragmentation of the GC/MS signals of the reaction mixture consisting of 0.56 mol/L MM^{OMe}, 0.28 mol/L propionaldehyde, and 0.5 mol% $Cp^{*}Ge^{+}$ $B(C_6F_5)_4^{-}$ recorded after 24 h of reaction.

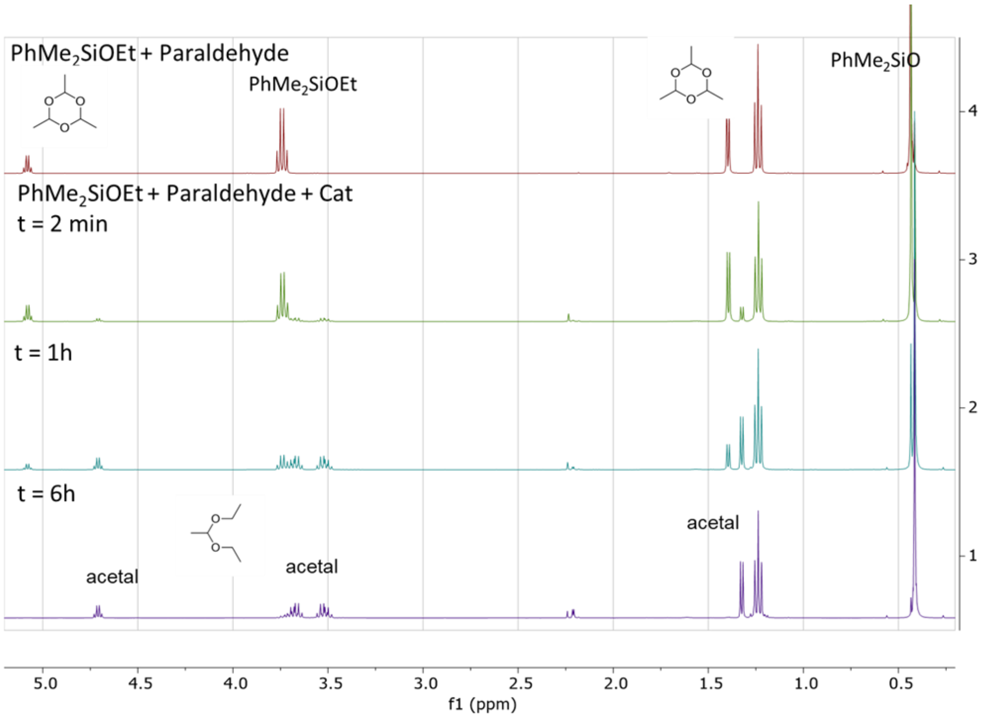

Figure S10. ^{1}H NMR spectra of the reaction mixture consisting of 0.56 mol/L of $PhMe_2SiOEt$ with 0.09 mol/L paraldehyde in the presence of 0.0028 mol/L $Cp^{*}Ge^{+}$ $B(C_6F_5)_4^{-}$ recorded before the addition of the catalyst, 2 min, 60 min, and 360 min after the addition of the catalyst.

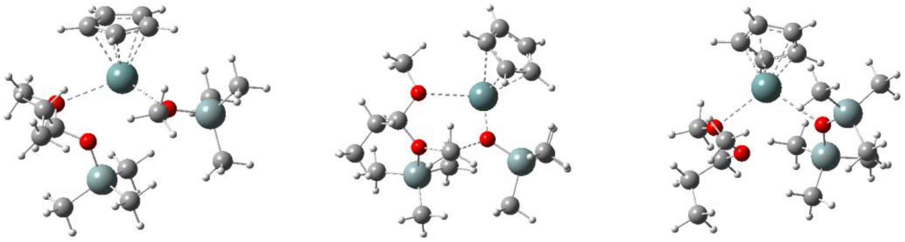

(**IV(1)**) (**IV-TS(1)**) (**V(1)**)

Figure S11. Calculated structures of the stationary points **IV(1) and V(1)** and the transition state, **IV-TS(1)**, (2nd stage, Variant 1) of the de-alkoxylation reaction catalyzed by $CpGe^{+}$. Colors of elements: white – H, grey – C, steel-grey – Si, darker steel-grey – Ge, red – O.

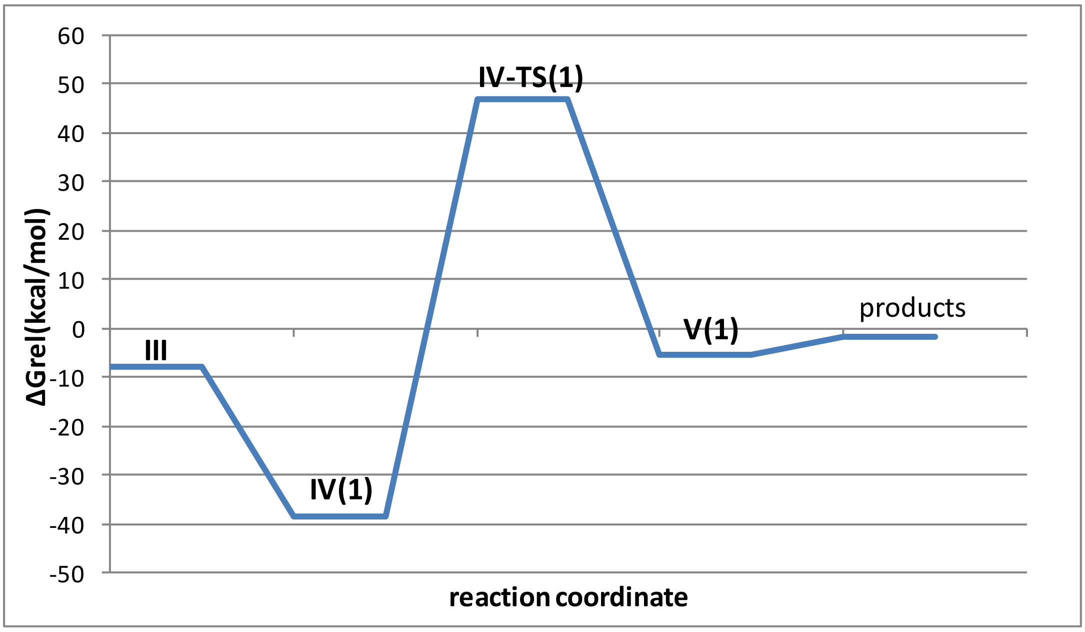

Figure S12. Calculated relative enthalpies and free energies (Kcal/mol) of stationary points along the reaction path of the mechanism involving the attack on Si center (2nd Stage, Variant 1).
